# A comprehensive investigation of the mRNA and protein level of ACE2, the putative receptor of SARS-CoV-2, in human tissues and blood cells

**DOI:** 10.7150/ijms.46695

**Published:** 2020-06-18

**Authors:** Yiliang Wang, Yun Wang, Weisheng Luo, Lianzhou Huang, Ji Xiao, Feng Li, Shurong Qin, Xiaowei Song, Yanting Wu, Qiongzhen Zeng, Fujun Jin, Yifei Wang

**Affiliations:** 1 Guangzhou Jinan Biomedicine Research and Development Center, Institute of Biomedicine, College of Life Science and Technology, Jinan University, Guangzhou 510632, PR China;; 2 Department of Obstetrics and Gynecology, The First Affiliated Hospital of Jinan University, Guangzhou 510632, PR China;; 3 Key Laboratory of Virology of Guangzhou, Jinan University, Guangzhou 510632, P.R, China;; 4 Key Laboratory of Bioengineering Medicine of Guangdong Province, Jinan University, Guangzhou 510632, P.R, China.

**Keywords:** SARS-CoV-2, Angiotensin-converting enzyme 2, Human tissues, Blood cells

## Abstract

The outbreak of pneumonia caused by SARS-CoV-2 posed a great threat to global human health, which urgently requires us to understand comprehensively the mechanism of SARS-CoV-2 infection. Angiotensin-converting enzyme 2 (ACE2) was identified as a functional receptor for SARS-CoV-2, distribution of which may indicate the risk of different human organs vulnerable to SARS-CoV-2 infection. Previous studies investigating the distribution of ACE2 mRNA in human tissues only involved a limited size of the samples and a lack of determination for ACE2 protein. Given the heterogeneity among humans, the datasets covering more tissues with a larger size of samples should be analyzed. Indeed, ACE2 is a membrane and secreted protein, while the expression of ACE2 in blood and common blood cells remains unknown. Herein, the proteomic data in HIPED and the antibody-based immunochemistry result in HPA were collected to analyze the distribution of ACE2 protein in human tissues. The bulk RNA-seq profiles from three separate public datasets including HPA tissue Atlas, GTEx, and FANTOM5 CAGE were also obtained to determine the expression of ACE2 in human tissues. Moreover, the abundance of ACE2 in human blood and blood cells was determined by analyzing the data in the PeptideAtlas and the HPA Blood Atlas. We found that the mRNA expression cannot reflect the abundance of ACE2 factor due to the strong differences between mRNA and protein quantities of ACE2 within and across tissues. Our results suggested that ACE2 protein is mainly expressed in the small intestine, kidney, gallbladder, and testis, while the abundance of which in brain-associated tissues and blood common cells is low. HIPED revealed enrichment of ACE2 protein in the placenta and ovary despite a low mRNA level. Further, human secretome shows that the average concentration of ACE2 protein in the plasma of males is higher than those in females. Our research will be beneficial for understanding the transmission routes and sex-based differences in susceptibility of SARS-CoV-2 infection.

## Introduction

The 2019 novel coronavirus disease (COVID-19) is highly infectious 1, 2, outbreak of which has been announced as a global pandemic by the World Health Organization on 11 March 2020. The pathogen contributed to COVID-19 is Severe Acute Respiratory Syndrome Coronavirus 2(SARS-CoV-2), a sister of SARS-CoV 3, 4. As showed by the Center for Systems Science and Engineering at Johns Hopkins University (latest updated at 05/20/2020), the global cumulative number of confirmed cases has reached 5,019,609, with 1,983,479 cured cases and 325,855 deaths 5. However, there were currently no effective drugs and vaccines against SARS-CoV-2, which was partly limited by the lack of recognition of the mechanism of SARS-CoV-2 infection. Previous studies had reported that SARS-CoV-2 used angiotensin-converting enzyme 2(ACE2) as the host receptor, but not other coronavirus receptors such as aminopeptidase N and dipeptidyl peptidase 4 3, 6. The RNA binding domain of SARS-CoV-2 spike protein binds to ACE2 with a 20-30-fold higher affinity than SARS-CoV 6-9, which may contribute to the rapid transmission of COVID-19. Therefore, the distribution of ACE2 in human tissues may indicate the susceptibility of different human organs to SARS-CoV-2 infection 10. Some studies explored the heterogeneity of ACE2 expression in specific tissue at the single-cell level 10-13. Also, there were several studies using transcriptome data to analyze the distribution of ACE2 mRNA in human tissues 14-17. However, all these researches only analyzed the mRNA level of ACE2, while failed to determine the distribution of ACE2 protein. Indeed, given the complex composition of a tissue, the result of single-cell RNA-sequencing also cannot reflect the average abundance of ACE2 in the whole tissues. Further, ACE2 is a membrane and secreted protein, while the abundance of ACE2 in blood and common blood cells remains uncertain.

To obtain the accurate distribution of ACE2 in human tissues, we comprehensively investigated the level of ACE2 mRNA and protein across human tissues and blood using public transcriptome datasets, mass spectrum-based tissues proteomic and secretome, and antibody-based immunochemistry (IHC). Our study would have implications for understanding the transmission routes of SARS-CoV-2 as well as the pathogenesis and future treatment for SARS-CoV-2.

## Results

### The mRNA expression of ACE2 in normal human tissues using different separate public datasets

**Figure 1:**
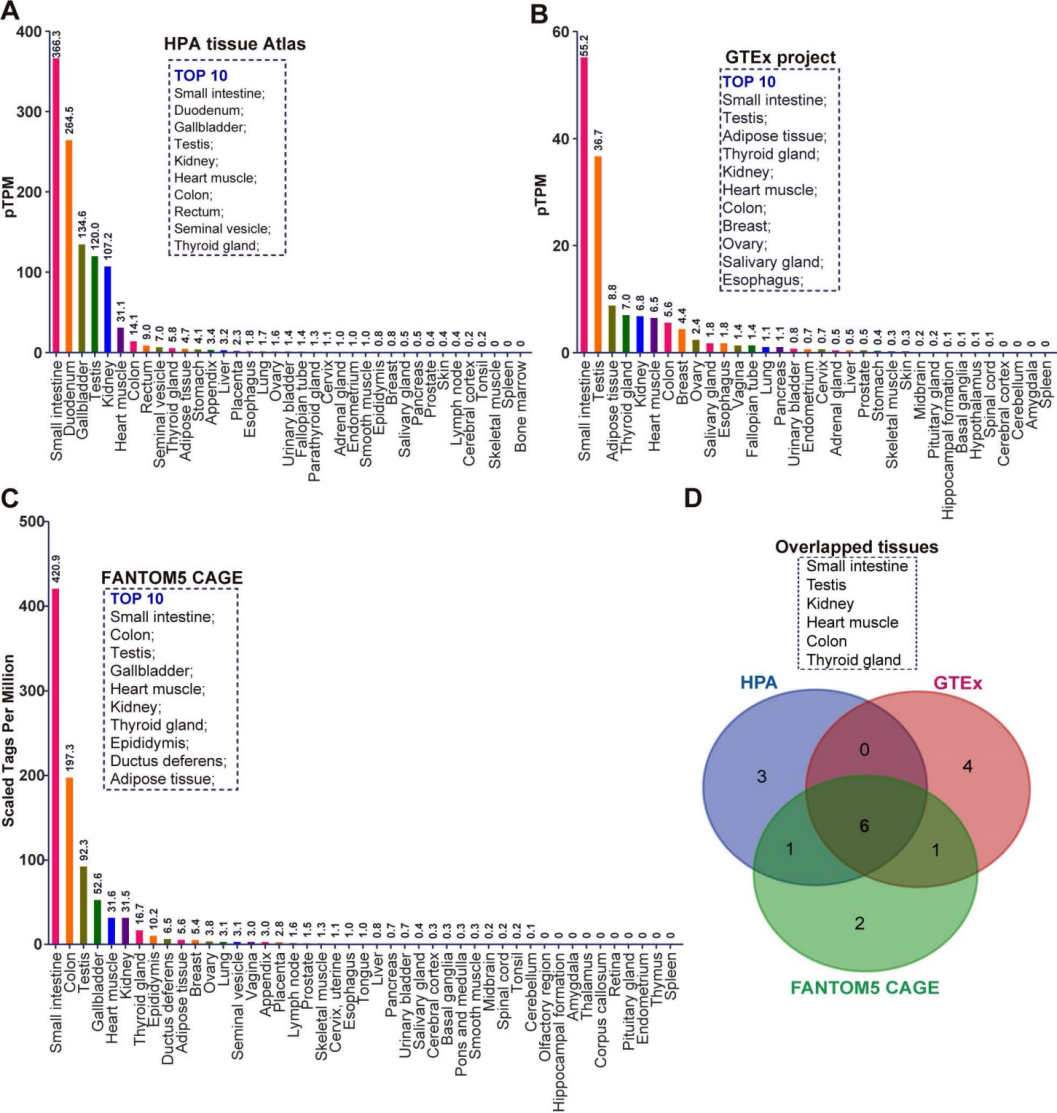
** The mRNA expression of ACE2 in normal human tissues in different separate public datasets. (A)** Bar plot of ACE2 mRNA expression across normal human tissues and organs based on the RNA-sequencing datasets in HPA tissue Atlas. The level of ACE2 mRNA was represented by the mean protein-coding transcripts per million (pTPM). The top10 tissues with highest abundance of ACE2 mRNA were also indicated; **(B)** Bar plot of the transcript abundance of ACE2 across normal human tissues and organs based on the RNA-sequencing datasets in GTEx project. The level of ACE2 mRNA was represented by the mean pTPM. The top10 tissues with highest abundance of ACE2 mRNA were also indicated; **(C)** Bar plot of the transcript abundance of ACE2 across normal human tissues and organs based on the FANTOM5 CAGE dataset. The level of ACE2 mRNA was reflected by the normalized Tags Per Million for ACE2, which were calculated by HPA; The top10 tissues with highest abundance of ACE2 mRNA were also indicated; **(D)** Venn diagram analysis (https://bioinfogp.cnb.csic.es/tools/venny/index.html) for obtaining the overlapped tissues with top10 highest level of ACE2 mRNA from the result of (A), (B), and (C).

To comprehensively investigate the mRNA expression of ACE2 in human tissues, we collected the transcriptome datasets in three public databases, including the Tissue Atlas of Human Protein Atlas (HPA), Genotype-Tissue Expression (GTEx), and Functional Annotation of Mammalian Genomes 5 (FANTOM5) Cap Analysis of Gene Expression (CAGE) 18-22. These data were separately analyzed according to the description in Materials and Methods. Given the samples of each tissue maybe come from the different individuals, all RNA-seq tissue data were present as mean protein-coding transcripts per million (pTPM), corresponding to mean values of the different individual samples from each tissue. As indicated by the result of HPA, ACE2 mRNA can be virtually detected in many tissues (**Figure 1A**). The top 10 tissues with the highest abundance of ACE2 mRNA are small intestine (pTPM: 366.3), duodenum (264.5), gallbladder (134.6), testis (120.0), kidney (107.2), heart muscle (31.1), colon (14.1), rectum (9.0), seminal vesicle (7.0), and thyroid gland (5.8) (**Figure 1A**). Most belonged to gastrointestinal tract-associated tissues or organs including the small intestine, duodenum, colon and rectum. Several were male reproductive system-associated organs, including testis and seminal vesicle, in accordance with the findings of previous publication 16. As showed by the result of GTEx RNA-seq data, the top 10 tissue with the highest abundance of ACE2 mRNA were small intestine (pTPM:55.2), testis (36.7), adipose tissue (8.8), thyroid gland (7.0), kidney (6.8), heart muscle (6.5), colon (5.6), breast (4.4), ovary (2.4), salivary gland (1.8), and esophagus (1.8) (**Figure 1B**). Consistent with the result of HPA, a relatively high abundance of ACE2 mRNA was observed in gastrointestinal tract-associated organs, i.e. small intestine and colon. The breast and female reproduction system-associated organs including breast and ovary also rank in the top 10, which is distinct from HPA (**Figure 1B**). The vagina also expressed a low level of ACE2 mRNA with a pTPM of 1.4, which was close to the salivary gland and esophagus (**Figure 1B**). The mRNA expression of ACE2 in tissue obtained from the FANTOM5 project was reported as scaled tags per million. The top 10 tissue with the highest abundance of ACE2 mRNA in FANTOM5 project included small intestine (scaled tags per million: 420.9), colon (197.3), testis (92.3), gallbladder (52.6), heart muscle (31.6), kidney (31.5), thyroid gland (16.7), epididymis (10.2), ductus deferens (6.5), and adipose tissue (5.6) (**Figure 1C**). Of note, despite small intestine and colon rank in Top2 tissues with the highest abundance of ACE2 mRNA, the male reproduction system-associated organs also occupied three of ten, i.e., testis, epididymis, and ductus deferens (**Figure 1C**). Given the minor difference between these three data sets, we analyzed the overlapped tissues among them using a Venn diagram. As demonstrated by the Venn diagram, there were six overlapped tissues with the top10 highest abundance of ACE mRNA, including testis, kidney, heart muscle, colon, and thyroid gland (**Figure 1D**).

### The distribution of ACE2 protein in human tissues using immunohistochemistry-based protein profiles and mass spectrometry-based proteomic dataset

**Figure 2:**
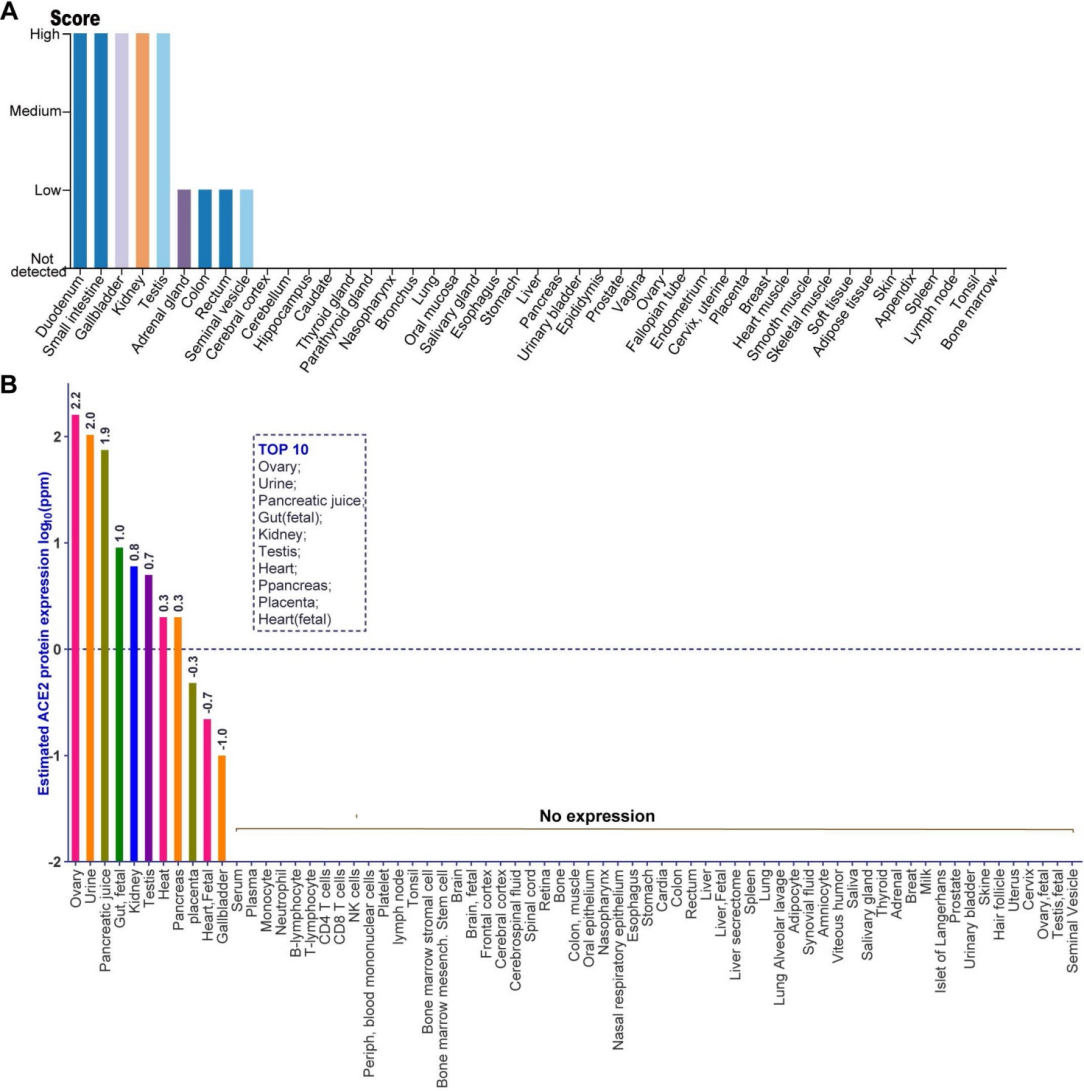
** The distribution of ACE2 protein in human tissues obtained by immunohistochemistry-based protein profiles and mass spectrometry (MS)-based proteomic dataset. (A)** The rough relative abundance of ACE2 in human tissues based on immunohistochemistry-base protein profiles, which was obtained from HPA. The antibody-based rough relative abundance was determined by combining the basic annotation and knowledge-based annotation. The basic annotation includes staining intensity, average positive staining area percentage, and subcellular localization. For the obscure samples, the knowledge annotation such as RNA level was introduced to assess the abundance of protein. The data no histogram meant no ACE2 expression in corresponding samples. Detailed information can be obtained from the Materials and Methods; **(B)** The protein expression of ACE2 based on normalized abundances in 69 normal human anatomical entities. The ppm values of ACE2 for each anatomical entity were calculated to present as expression value according to the description in Materials and Methods. The expression values were drawn on a root scale, an intermediate between log and linear scales. Top10 tissues with highest abundance of ACE2 protein were also indicated. The data no labeling meant no ACE2 expression in corresponding samples.

Given the level of ACE2 mRNA cannot represent the abundance of a functional protein, we investigated the distribution of ACE2 protein in human tissues through analyzing the immunohistochemistry data from normal tissue in HPA and the public mass spectrometry (MS)-based proteomics data in Human Integrated Protein Expression Database (HIPED). The antibody-based protein profiles were analyzed by HPA, which based on basic annotation and knowledge-based annotation, to describe the rough relative abundance of ACE2 proteins in various tissues. Detailed information can be obtained from the Materials and Methods. As indicated by the quantitative result of immunohistochemistry, there was a high abundance of ACE2 protein in five tissues, including the small intestine, duodenum, gallbladder, kidney, and testis, (**Figure 2A**). By contrast, the adrenal gland, colon, rectum, and seminal vesicle expressed a low level of ACE2 protein (**Figure 2A**). Moreover, ACE2 protein was not detected in other tissues including the cerebral cortex, cerebellum, hippocampus, caudate, thyroid gland, nasopharynx, bronchus, lung, oral mucosa, salivary gland, esophagus, stomach, liver, pancreas, and urinary bladder et.al, based on the rough quantitative result of immunohistochemistry (**Figure 2A**). Next, we analyzed the distribution of ACE2 protein in human tissues using the MS-based proteomics dataset in HIPED. The level of ACE2 protein was calculated based on the spectral counts. The quantitative results suggested that the ACE2 protein was enriched in the ovary (log_10_ppm: 2.2), urine (2.0), pancreatic juice (1.9), gut (fetal) (1.0), kidney (0.8), testis (0.7), heart (0.3), pancreas (0.3), placenta (-0.3), heart (fetal) (-0.7), and gallbladder (-1.0), which were ordered by the abundance of ACE2 protein (**Figure 2B**). In particular, ACE2 protein was positively differentially expressed in that entity in the ovary, urine, and pancreatic juice, as defined by the quantitative result in HIPED (**Figure 2B**). However, the unique peptides of ACE2 protein cannot be tested in both the fetal ovary and testis (**Figure 2B**). Moreover, the ACE2 protein also tested negative in human common blood cells, including monocyte, neutrophil, B-lymphocyte, T-lymphocyte, CD4 T-cells, CD8 T-cells, NK cells, and peripheral blood mononuclear cells (**Figure 2B**).

### The expression of ACE2 in human plasma and common blood cells

**Figure 3:**
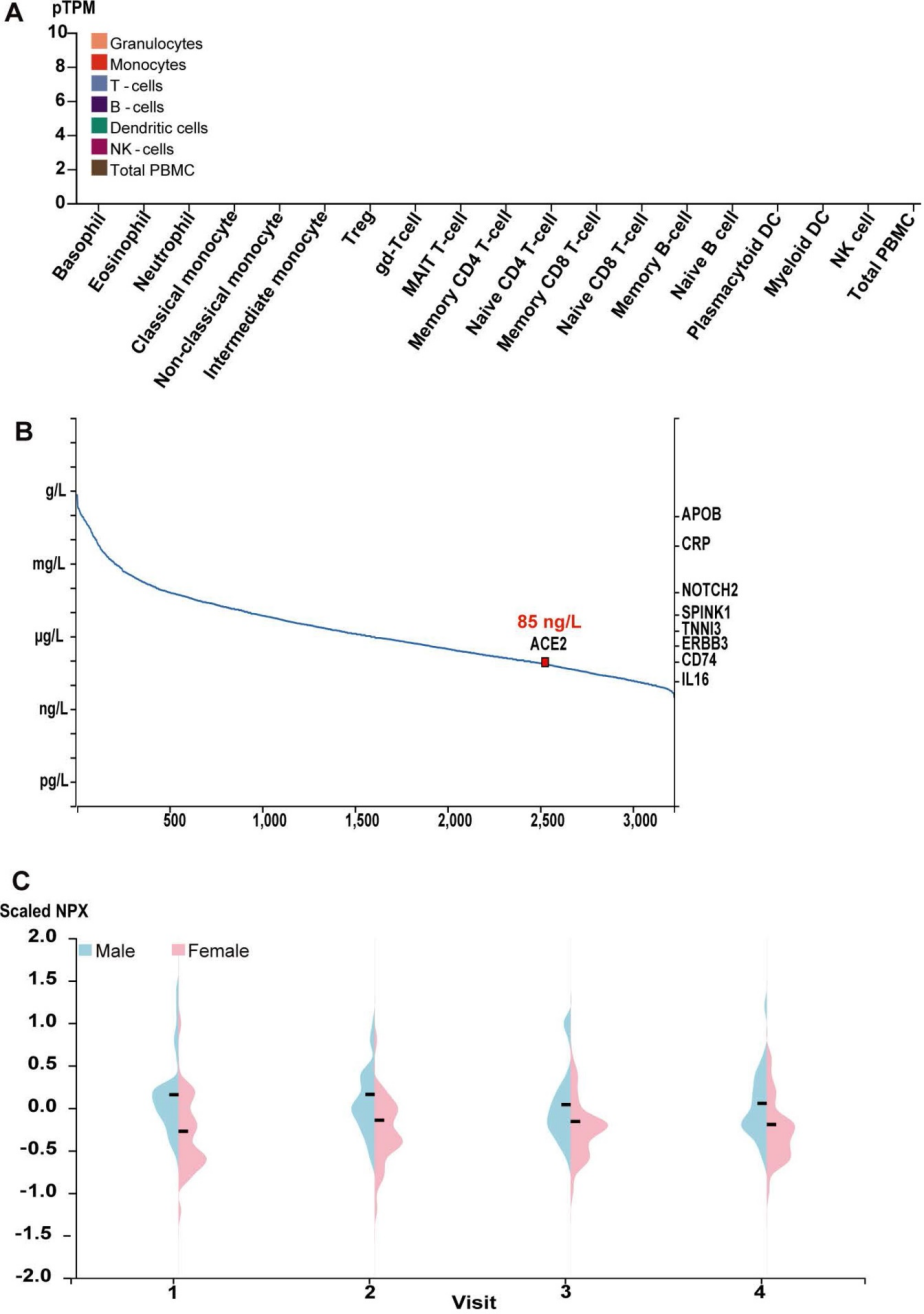
** The level of ACE2 mRNA in common blood cells and the abundance of ACE2 protein in human plasma. (A)** Bar plot of the transcript abundance of ACE2 across common blood cells. The level of ACE2 mRNA was represented by the mean pTPM; The data no labelling meant no ACE2 expression in corresponding samples; **(B)** The concentration of ACE2 in human plasma based on the spectral counts in the proteomics of HIPED, which was obtained from HPA. Other proteins in right Y axis were also labeled in the position corresponding to their concentration; **(C)**Violin plots showing the distribution of levels of ACE2 protein in plasma across the individuals for four visits during one years in females and males. Protein expression levels were represented as Normalized Protein Expression (NPX).

Give ACE2 is a membrane and secreted protein, we analyzed the level of ACE2 mRNA in the common blood cells using the public transcriptome data for the common human blood cells sorted by the flow cytometry in HPA Blood Atlas 23, 24. Consistent with the proteomic data for ACE2 protein, ACE2 mRNA was also tested negative in virtually all blood cells, including the basophil, eosinophil, neutrophil, classical monocyte, non-classical monocyte, intermediate monocyte, regulatory T-cells, memory CD4 T-cell, naive CD4 T-cell, memory CD8 T-cell, naive CD8 T-cell, memory B-cell, naive B-cell, NK cell and total PBMC et al., as indicated by the quantitative results (**Figure 3A**). Indeed, such results were further supported by the transcriptome in other different databases, including Monaco scaled dataset and Schmiedel dataset 25, 26 **(Supplement Figure 1)**. We also obtained the abundance of ACE2 protein from human blood using MS-based proteomics in PeptideAtlas 27-29 and proximity extension assays-based protein profiling in HPA blood Atlas 23, 24. Based on the spectral counts of MS-based proteomics in the PeptideAtlas, the concentration of ACE2 protein in plasma approximately reached to 85 ng/L (**Figure 3B**). Further, as showed by the quantity results of proximity extension assays-based protein profiling, the average concentration of ACE2 protein in plasma from male showed a minor higher than those from female across four visits during one year (**Figure 3C**).

## Discussion

With the global outbreak of COVID-19, the development of the drugs against SARS-CoV-2 had become an urgent work. Accomplishment of the virus life cycle largely depends on host factors; therefore, targeting the virus-host interactions and host cellular mechanisms are promising treatment options 30. On the theory, the agents with the ability to target any step in the virus life cycle can be designed as antiviral drugs. Cell entry is the first step of cross-species transmission of the virus, of which for SARS-CoV-2 was mediated by the spike proteins on its surface to bind to the ACE2 receptor 3, 6-9. Based on the strategy of targeting ACE2, several scientists have screened potential anti-SARS-CoV-2 drugs 31, 32. Therefore, the comprehensive investigation of ACE2 expression in human tissues has implications for understanding the transmission routes of SARS-CoV-2 and the development of anti-COVID-19 drugs. Previous studies had investigated the distribution of ACE2 mRNA in human tissues and explored the expression heterogeneity among specific tissue using single-cell RNA-sequencing but with a limited size of samples and a lack of determining the protein level of ACE2 10-17. However, given the great heterogeneity among humans, the transcriptome and proteomic dataset with a larger number of samples and a wider range of tissues such as blood cells should be collected to analyze. Indeed, a prior deep proteome and transcriptome of 29 healthy human tissues suggested a strong difference between mRNA and protein quantities and that protein expression was often more stable across tissues than that of transcripts 33. Therefore, protein is a more accurate indicator than the mRNA of reflecting the abundance of ACE2. In this study, we made a comprehensive investigation of the mRNA and protein expression of ACE2 in human tissues using public transcriptome, proteomic datasets, and antibody-based protein profiles. Given blood immune cells are also crucial for combating virus infection, we analyzed the expression of ACE2 mRNA and protein in common blood cells and plasma.

Despite all these databases utilized in this study based on RNA-seq, there is a minor difference among the transcriptome result obtained from different databases, which may be caused by tissue source, technical artifacts inherent in the respective methodologies, and gene model annotation issues among them. In detail, the RNA data in HAP based on surgically removed tissues^18^, while those in GTEx based on postmortem samples 19, 20. Moreover, the mRNA without polyadenylation tails are excluded in HPA, leading to the absence of many histone genes, while which are present in the FANTOM. Further, the cap analysis gene expression peaks mapping more than 500 base pairs from the transcription start site are absent in the FANTOM. Also, the peaks mapping more than one location on the genome are removed from FANTOM 34.

Of note, we found that the lung expresses a low level of ACE2 in both mRNA and protein, which seems to be controversial with the lung as the main tissue with the typical symptoms in response to SARS-CoV-2 infection. Such a result can be explained by several reasons as follows. Specifically, and in particular, as revealed by prior single-cell RNA-sequencing, ACE2 expression positive is only observed in a small population (approximately 1%) of type II alveolar cells, while the remaining cell population in lung express a low level of ACE2 10, 11, which supported previous immunohistochemistry 35. However, SARS-CoV-2 infection remarkably induces the expression of ACE2, as an interferon-stimulated gene, in human airway epithelial cells 36, 37. Therefore, the type II alveolar cells would represent a basic target of SARS-CoV-2 in the lung. The infection of SARS-CoV-2 in type II alveolar cells upregulates the level of ACE2 in the lung and thereby further facilitates the infection of SARS-CoV-2. Indeed, we also cannot exclude the possibility that SARS-CoV-2 used other unknown factors as a receptor that may be highly expressed in lung, especially given that previous study had revealed that both TMPRSS2 and CD147 also partly mediated the cell entry of SARS-CoV-2 32, 38. Also, a host restriction factor that lowly expressed in the lung for SARS-CoV-2 cell entry needs to be considered. Indeed, in accordance with previous research 39, we noted that A549 lung alveolar cells expressed a low level as indicated by HPA Cell Atlas (data not show), implying the cell line is not an ideal model to study SARS-CoV-2 *in vitro*. The *in vitro* model of SARS-CoV-2 infection should be established using A549 with exogenous ACE2 or other primary lung-derived cells, such as normal human bronchial epithelial cells 39.

Both testis and kidney tissues expressed a high level of ACE2 mRNA and protein, which is consistent with previous publications 13, 16. Such results may explain the damage of testis and the impairment of male gonadal function caused by SARS-CoV-2 40. Moreover, both antibody-based IHC and tissue transcriptome data also showed a high abundance of ACE2 in the small intestine, which is supported by a single-cell transcriptome of revealing that the digestive system may be an important route of SARS-CoV-2 transmission 12. The high expression level of ACE2 in the gastrointestinal tract may explain why most of COVID-19 patients show gastrointestinal symptoms in the early stage of the infection 41. However, the proteomic data of the small intestine is not available in the HIPED. By contrast, some tissues harbor with a high abundance of ACE2 mRNA but show a low level of ACE2 protein. For example, ACE2 mRNA can be detected in the bladder, which is consistent with a previous study 13, while no ACE2 protein was observed in urinary bladder as indicated by the quantitative result of proteomic data and antibody-based IHC. Given there was a divergence toward the potential of intrauterine vertical transmission in women who develop COVID-19 pneumonia during pregnancy 42-44, we also paid attention to the expression of ACE2 in the female reproduction-associated tissues. Of note, the quantitative result of transcriptome supported that ovary virtually did not express ACE2, while ovary was the organ with the highest level of ACE2 protein as indicated by the proteomic data. The placenta also expressed a high level of ACE2 protein. Based on these results, the intrauterine vertical transmission potential of SARS-CoV-2 cannot be underestimated despite uterus was tested negative for ACE2 protein.

Of note, all the transcriptome in different database revealed no ACE2 mRNA and protein in the common blood cells, including basophil, eosinophil, neutrophil, classical monocytes, non-classical monocyte, Treg, gd-T cell, MAIT T-cell, memory CD4 T-cell, naïve CD4 T-cell, memory B-cell, naïve B-cell, plasmacytoid DC, myeloid DC, NK cell, and total PBMC, suggesting the potential of resistance of immune cell against SARS-CoV-2. However, these results did not suggest that viral particles cannot survival from blood because the public human secretome suggested the concentration of ACE2 protein in plasma is approximately 85 ng/L. Indeed, according to the description in the latest New Coronavirus Pneumonia Prevention and Control Program published by the National Health Commission of China, the nucleotides of SARS-CoV-2 can be tested from the blood sample of patients. SARS-CoV-2 infection also caused a remodeling myeloid in severe COVID-19 patients 45. Interestingly, a prior study reported that COVID-19 susceptibility seems to be related to blood group, whereas whether such results are associated with the level of ACE2 remains uncertain 46. Further, although our result found that there was virtually no ACE2 mRNA and protein in the central nervous system (CNS), including the cerebral cortex, brain, cerebrospinal fluid, and cerebellum, the possibility of CNS infection of SARS-CoV-2 cannot be neglected. Indeed, accumulating evidence supported the neuroinvasive potential of SARS-CoV2 47, 48. There are also several studies have shown that ACE2 enzymatic activity can be detected in human brain tissue and CSF samples 49, indicating that ACE2 is expressed and functional in the CNS of humans. The way the tissue is fixed or processed can affect the result of IHC or proteomics of these databases. Specifically, there is no need for an autopsy to study human blood. By contrast, unlike the data from blood cells, for tissue like brain, it required autopsy study.

In summary, our study reveals that the tissue distribution of ACE2 mRNA and protein might differ and their correlation is complex. However, our study was limited by only analyzed public datasets with a lack of confirmation by experiments. Nevertheless, our study would be beneficial for understanding the risk of different human organs vulnerable to SARS-CoV-2 infection.

## Materials and Methods

### Transcriptome sequencing data for human tissues acquisition and analysis

To obtain the comprehensive information regarding the transcriptome of human tissues, we collected the transcriptome from three public databases, including the Human Protein Atlas (HPA) tissues atlas, Genotype-Tissue Expression (GTEx) project, and Functional Annotation of Mammalian Genomes 5 (FANTOM5) project. All these databases update irregularly and the version of them we analyzed is the latest. In detail, the HPA shows the expression of human proteins across tissues and organs based on deep RNA-sequencing from 37 major different normal tissue types 18. The mRNA data in the HPA tissue Atlas provide quantitative data on the average gene expression within an entire tissue to estimate the transcript abundance of each protein-coding gene. The HPA integrates RNA and protein expression data corresponding to approximately 80% of the human protein-coding genes with access to the primary data for both the RNA and the protein analysis on an individual gene level. The GTEx project includes genotype data from approximately 714 donors and 11688 RNA-seq samples across 53 tissues 19, 20. RNA-seq data from 36 of their tissue types were mapped based on RSEMv1.2.22 (v7) and the resulting TPM values have been included in HPA for all corresponding genes. Both HPA and GTEx RNA-seq tissue of the protein-coding gene is reported as mean pTPM (protein-coding transcripts per million), corresponding to mean values of the different individual samples from each tissue. The FANTOM5 project provides comprehensive expression profiles using Cap Analysis of Gene Expression (CAGE) 21, 22, which is based on a series of full-length cDNA technologies developed in RIKEN. CAGE data for 60 of their tissues were obtained from the FANTOM5 repository and mapped to ENSEMBL. The normalized Tags Per Million for each gene were calculated in HPA.

### HPA immunohistochemistry data for human tissues acquisition and analysis

The HPA Tissue Atlas shows the expression of human proteins across tissues and organs based on immunohistochemistry on tissue microarrays containing 44 different tissues 18, 50. Annotated protein expression profiles were obtained by single antibodies or independent antibodies (two or more independent antibodies with non-overlapping epitopes on the same protein). For independent antibodies, the immunohistochemical data from all the different antibodies were taken into consideration. To obtain a comprehensive overview of protein expression patterns in normal human tissues, the basic annotation was combined with knowledge-based annotation to determine the rough relative abundance of proteins in these tissues as calculated by HPA tissue Atlas. Basic annotation parameters include an evaluation of i) staining intensity (negative, weak, moderate or strong), ii) fraction of stained cells (<25%, 25-75% or >75%) and iii) subcellular localization (nuclear and/or cytoplasmic/membranous). Knowledge-based annotation was achieved by stringent evaluation of immunohistochemical staining pattern, RNA-seq data from internal and external sources and available protein/gene characterization data, with special emphasis on RNA-seq. All immunohistochemical images are available and the annotation data can be found under primary data in HPA.

### The proteomics datasets of Human Integrated Protein Expression Database (HIPED) acquisition and analysis

HIPED is an integrated proteomics platform residing within GeneCards, which involved 69 normal anatomical entities (tissues, cells, and fluids) from four databases including ProteomicsDB, MOPED, PaxDb, and MaxQB 51. The ppm protein values were calculated for each sample, if not provided so by data sources. The intensity-based absolute quantification (iBAQ) expression values were divided by the sum of values of each sample and multiplied by 1,000,000. iBAQ is a proxy for protein abundance levels 52. For all samples, data was gene centrically aggregated by summing expression values of all isoforms for each gene. Samples from similar tissues were averaged, using geometric mean. For better visualization of graphs, expression values are drawn on a root scale, which is an intermediate between log and linear scales 51. The protein expression images present a protein expression vector for each gene, based on normalized abundances in 69 normal human anatomical entities.

Protein differential expression provides a list of anatomical entities for which a gene is positively differentially expressed, based on the 69 integrated normal proteomics datasets in HIPED. Genes with fold change value >6 and protein abundance value >0.1 PPM in an anatomical entity are defined as positively differentially expressed in that entity. Fold change values were calculated as the ratio between the tested dataset protein abundance and the average of all datasets.

### The transcriptome, MS-based proteomics datasets, and antibody-based immune assays of the Human Blood Atlas acquisition and analysis

The Blood Atlas contains single cell type information on genome-wide RNA expression profiles of human protein-coding genes covering 18 cell types obtained by Fluorescence-Activated Cell Sorting 23. These cells include various B-cells, T-cells, NK-cells, monocytes, and dendritic cells. The RNA expression for each gene was analyzed by the online tools resided in HPA blood Atlas. The public transcriptome datasets from Schmiedel B.J. 25 and Monaco G. 26 were also collected to analyze the mRNA expression of ACE2 in human common blood cells. Give ACE2 is a membrane and secreted protein in the blood we further analyzed its abundance in the human blood using public MS-based proteomics in PeptideAtla 27-29 and HPA blood Atlas 24. An analysis of the proteins detected in human blood was presented with an estimation of the respective protein concentrations determined with MS-based proteomics and proximity extension assays-based protein profiling. HPA Blood Atlas provides the protein concentration in plasma based on proximity extension assays (Olink) for a longitudinal wellness study covering 86 individuals with four visits during one year at three months intervals. Protein expression levels are reported as Normalized Protein Expression (NPX).

## Supplementary Material

Supplementary figure S1.

## Abbreviations

SARS-CoV-2: Severe Acute Respiratory Syndrome Coronavirus 2
COVID-19: Coronavirus Disease 2019
ACE2: Angiotensin-converting enzyme 2
SARS: Severe Acute Respiratory Syndrome
GTEx: Genotype-Tissue Expression
FANTOM5 CAGE: Functional Annotation of The Mammalian Genome Cap Analysis of Gene Expression
HPA: Human Protein Atlas
NPX: Normalized Protein Expression
