# Supplementary material for: A comprehensive investigation of the mRNA and protein level of ACE2, the putative receptor of SARS-CoV-2, in human tissues and blood cells
Source: Int J Med Sci. 2020 Jun 18;17(11):1522–31. doi: 10.7150/ijms.46695 (PMC7359402; doi:10.7150/ijms.46695)

Supplemental Figure 1. The level of ACE2 mRNA in common blood cells in the transcriptome datasets from Monaco scaled dataset and Schmiedel dataset.

(A) Bar plot of the transcript abundance of ACE2 across common blood cells obtained from the quantification of *Monaco* scaled dataset. The level of ACE2 mRNA was represented by the mean pTPM; The data no labelling meant no ACE2 expression in corresponding samples;

(B) Bar plot of the transcript abundance of ACE2 across common blood cells obtained from the quantification of *Schmiedel* dataset. The level of ACE2 mRNA was represented by the mean pTPM; The data no labelling meant no ACE2 expression in corresponding samples.

A

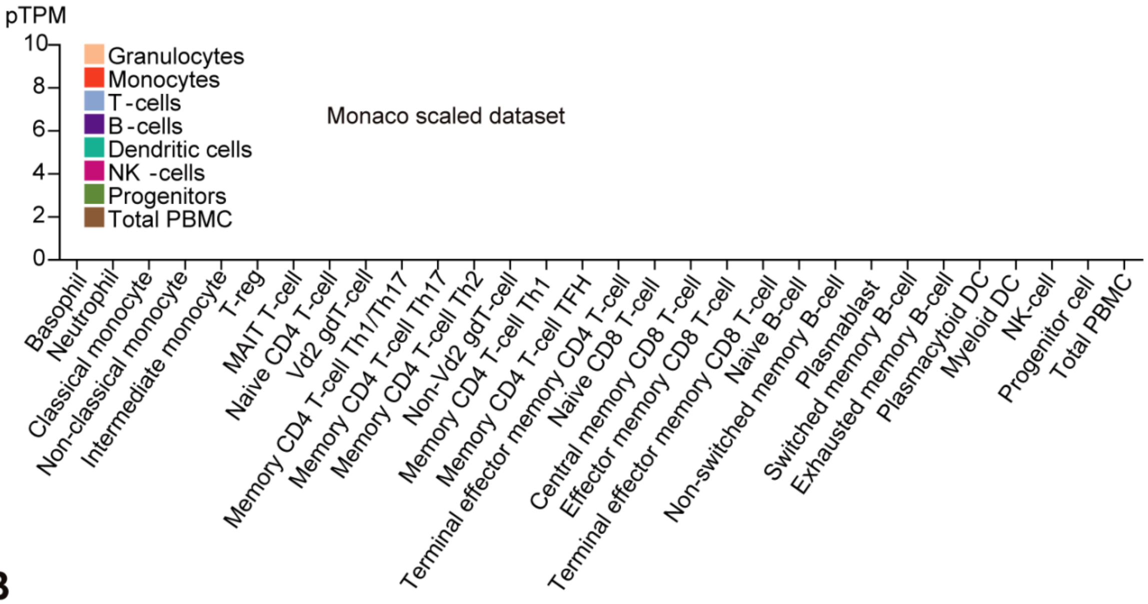

B

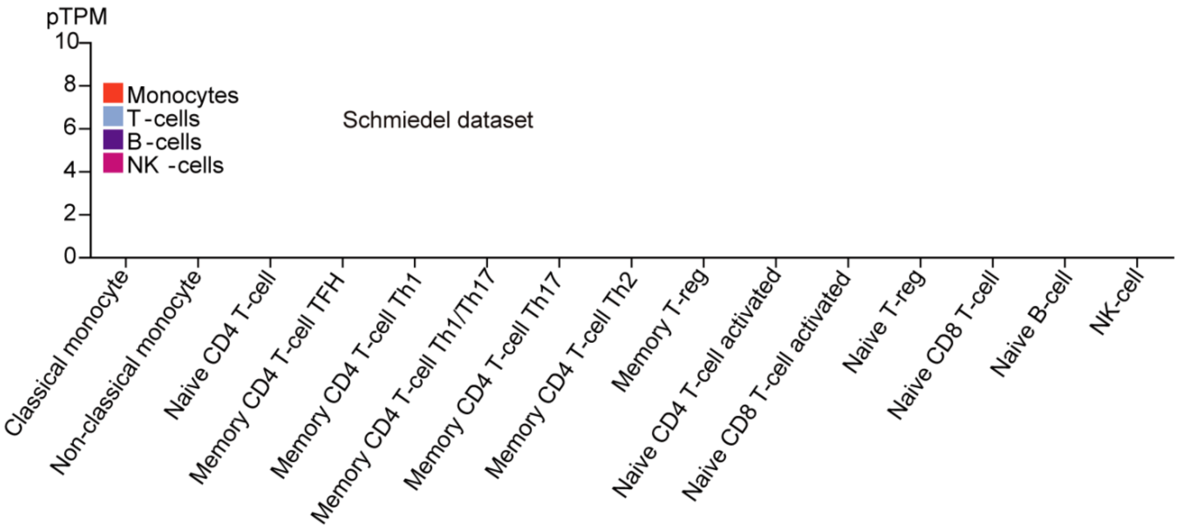

Supplement: Supplementary file 1 — Supplementary figure S1. [file ijmsv17p1522s1.pdf]
